# Supplementary material for: Contrasting patterns of evolutionary constraint and novelty revealed by comparative sperm proteomic analysis in Lepidoptera
Source: BMC Genomics. 2017 Dec 2;18:931. doi: 10.1186/s12864-017-4293-2 (PMC5712127; doi:10.1186/s12864-017-4293-2)
Supplement: Supplementary file 4 — Phylogenetic Results- Phylogeny exhibiting the evolutionary relationship of the thirteen insect species utilized in this study. (PDF 129 kb) [file 12864_2017_4293_MOESM4_ESM.pdf]

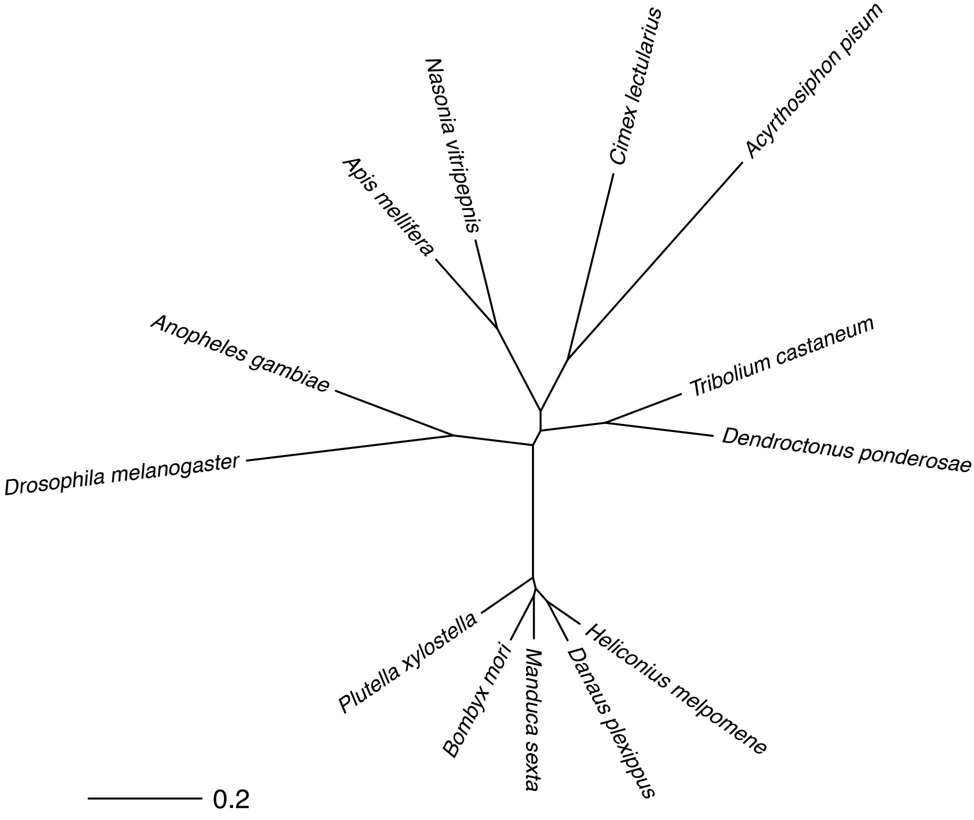

Additional File 4. Phylogenetic relationships among 13 insect taxa used for comparative genomic analysis in this study. The scale bar represents expected amino acid substitutions per site.
